# The 21-Gene Recurrence Score in Clinically High-Risk Lobular and Ductal Breast Cancer: A National Cancer Database Study

**DOI:** 10.1245/s10434-022-12065-3

**Published:** 2022-07-09

**Authors:** Mary Kathryn Abel, Amy M. Shui, A. Jo Chien, Hope S. Rugo, Michelle Melisko, Frederick Baehner, Rita A. Mukhtar

**Affiliations:** 1 School of Medicine, University of California, San Francisco, CA; 2 Department of Surgery, University of California, Box 1710, San Francisco, CA; 3 Department of Epidemiology and Biostatistics, University of California, San Francisco, CA; 4 Department of Medicine, San Francisco Helen Diller Family Comprehensive Cancer Center, University of California, San Francisco, CA; 5 Department of Pathology, University of California, San Francisco, CA

## Abstract

**Objective.:**

The aim of this study was to evaluate whether patients with invasive lobular carcinoma (ILC) are more likely to have discordant clinical and genomic risk than those with invasive ductal carcinoma (IDC) when using the 21-gene recurrence score (RS), and to assess overall survival outcomes of patients with 1–3 positive nodes and RS ≤25 with and without chemotherapy, stratified by histology.

**Methods.:**

We performed a cohort study using the National Cancer Database and included patients with hormone receptor-positive, HER2-negative, stage I–III invasive breast cancer who underwent 21-gene RS testing. Our primary outcome was rate of discordant clinical and genomic risk status by histologic subtype. Propensity score matching was used to compare 60-month overall survival in individuals with 1–3 positive nodes and RS ≤25 who did and did not receive chemotherapy.

**Results.:**

Overall, 186,867 patients were included in our analysis, including 37,685 (20.2%) patients with ILC. There was a significantly higher rate of discordant clinical and genomic risk in patients with ILC compared with IDC. Among patients with 1–3 positive nodes and RS ≤25, there was no significant difference in survival between those who did and did not receive chemotherapy in the IDC or ILC cohorts. Unadjusted exploratory analyses of patients under age 50 years with 1–3 positive nodes and RS ≤25 showed improved overall survival in IDC patients who received chemotherapy, but not among those with ILC.

**Conclusion.:**

Our findings highlight the importance of lobular-specific tools for stratifying clinical and genomic risk, as well as the need for histologic subtype-specific analyses in randomized trials.

Despite major advances in the treatment of breast cancer, personalizing therapy to reduce recurrence risk while minimizing harm remains a challenge. Recommendations for treatment such as adjuvant chemotherapy rely on multiple factors, including patient characteristics and overall health status, tumor features, and often molecular assays such as the 70-gene signature or the 21-gene recurrence score (RS).^1–3^ When patient and tumor factors suggest a high risk of recurrence, patients are deemed to have high ‘clinical’ risk and chemotherapy may be considered. Similarly, when molecular assays suggest a high risk of recurrence, tumors are considered to have high ‘genomic’ risk and patients with these high-risk tumors are predicted to benefit from chemotherapy.

However, when clinical and genomic risk are discordant, decisions about the efficacy of chemotherapy become more difficult. In the recently published RxPONDER trial, chemotherapy did not improve invasive disease-free survival in clinically high-risk postmenopausal patients with low genomic risk (based on RS ≤25), although benefit was seen in those who were premenopausal.^4^ These findings illustrate some of the challenges of treatment selection for those with discordant clinical and genomic risk.

We previously showed that such discordant risk status disproportionately affects patients with invasive lobular carcinoma (ILC) compared with invasive ductal carcinoma (IDC) when genomic risk is evaluated with 70-gene signature testing.^5^ As the second most common histologic subtype of breast cancer, ILC is thought to have a more indolent course but often presents at higher stages with considerable cumulative risk of late recurrence.^6–11^ We therefore sought to investigate rates of discordant clinical and genomic risk by histologic subtype in patients who underwent RS testing in the National Cancer Database (NCDB).

Specifically, we evaluated the following questions: (1) whether patients with ILC are more likely to have discordant clinical and genomic risk than those with IDC using the 21-gene RS; (2) whether use of chemotherapy differs by histologic subtype stratified by clinical and genomic risk category; and (3) whether patients with 1–3 positive nodes and RS ≤25 have different overall survival outcomes with or without adjuvant chemotherapy stratified by histologic subtype.

## METHODS

### Data Source and Study Cohort

Our study cohort consisted of patients in the NCDB, a national comprehensive clinical surveillance resource that represents over 70% of all newly diagnosed cancer cases in the US.^12,13^ Participant user files from 2010 to 2016 were used in our analysis, which included patients diagnosed between 2004 and 2016. Due to the de-identified nature of the public-access user files, the study did not require Institutional Review Board approval.

We limited our analysis to patients with invasive hormone receptor (HR)-positive and human epidermal growth factor receptor 2 (HER2)-negative tumors with an available 21-gene RS result. Tumors that were estrogen receptor (ER) and/or progesterone receptor (PR)-positive were considered to be HR-positive. We excluded patients with stage IV disease, those who received neoadjuvant therapy, individuals who did not undergo surgery for breast cancer, and those who were missing critical clinical information, including histologic subtype, tumor grade, number of positive lymph nodes on pathology, tumor size, and timing of chemotherapy or endocrine therapy.

### Clinical Measures and Outcomes

Our primary outcome was the rate of discordant clinical and genomic risk status by histologic subtype. We assigned clinical risk using a modified Adjuvant! Online calculator as described in the MINDACT trial; we also categorized patients by number of positive lymph nodes on surgical pathology (0, 1–3, or >3 nodes).^3,4,14–16^ The modified Adjuvant! Online risk score includes tumor size, tumor grade, and number of positive lymph nodes identified at surgery; those with a 92% or greater probability of breast cancer-specific survival at 10 years without chemotherapy were deemed to have low clinical risk.^17,18^ We assigned genomic risk using the 21-gene RS result, with RS >25 considered as high genomic risk and RS ≤25 considered as low genomic risk, consistent with RxPONDER. We defined ‘discordant risk’ as having either clinical high-risk with genomic low-risk status, or clinical low-risk with genomic high-risk status.

Histologic subtype was determined by defined codes in the NCDB. The ILC cohort included codes for ILC or mixed ILC/IDC (histology codes 8520 and 8524 if behavior was invasive). The IDC cohort comprised codes for IDC or invasive mammary carcinoma not otherwise specified (histology codes 8500, 8501, 8502, 8503, and 8523 if behavior was invasive). Menopausal status was approximated using age under 50 years (premenopausal) and over 50 years (postmenopausal) and was included as a predictor in our model. Additionally, the Charlson–Deyo Comorbidity Index was used as a marker of severity of comorbid conditions. The Charlson–Deyo Comorbidity Index is a weighted score from 0 to 3 derived from multiple comorbid conditions, including myocardial infarction, diabetes, and renal disease, with higher score reflecting more comorbid disease.^19–21^

### Statistical Analysis

Patient characteristics were described and differences between histologic subtype were evaluated using Chi-square tests for categorical variables and *t*-tests for continuous variables. Additionally, the frequency of patients in each of the four clinical and genomic risk categories (clinical high/genomic low, clinical low/genomic low, clinical high/genomic high, clinical low/genomic high) was described by histologic subtype, with Chi-square testing used to compare the clinical high/genomic low risk cohort compared with other risk categories combined and across all four risk categories. We also performed subgroup analyses evaluating concordance of genomic and clinical risk by categorical nodal status, histology, and age. Receipt of chemotherapy stratified by histology, nodal status, RS, and age was also assessed. All analyses were prespecified.

Finally, propensity score matching was used to compare 60-month overall survival in individuals who did and did not receive chemotherapy among patients with 1–3 positive nodes and RS ≤25, stratified by histology. A greedy, fixed 1:1 matching method was used and patients were matched by age at diagnosis in years, pathologic stage of disease (stage 2/3 vs. stage 1), and facility type. Weighted matched standardized differences and variance ratios for the propensity score model covariates were used to assess sample balance after matching. Acceptable balance was defined by a maximum of 0.2 for the absolute value of standardized difference and by values within the 0.5–2 range for variance ratio. While we planned a prespecified propensity score-matched analysis evaluating the relationship between chemotherapy and overall survival in the subgroup of women aged under 50 years, with 1–3 positive nodes and RS ≤25, there were too few patients to allow for matching. We therefore performed exploratory analyses evaluating overall survival with and without chemotherapy, using the log-rank test, in this subgroup of women, stratified by histology; we also used the Cox proportional hazards model to evaluate overall survival both unadjusted and adjusted for Charlson–Deyo Comorbidity Index.

Hypothesis tests were two-sided and the significance threshold was set to 0.05. Statistical analyses were performed using Stata 16 (StataCorp LLC, College Station, TX, USA) and SAS version 9.4 (SAS Institute, Cary, NC, USA).

## RESULTS

**FIG. 1:**
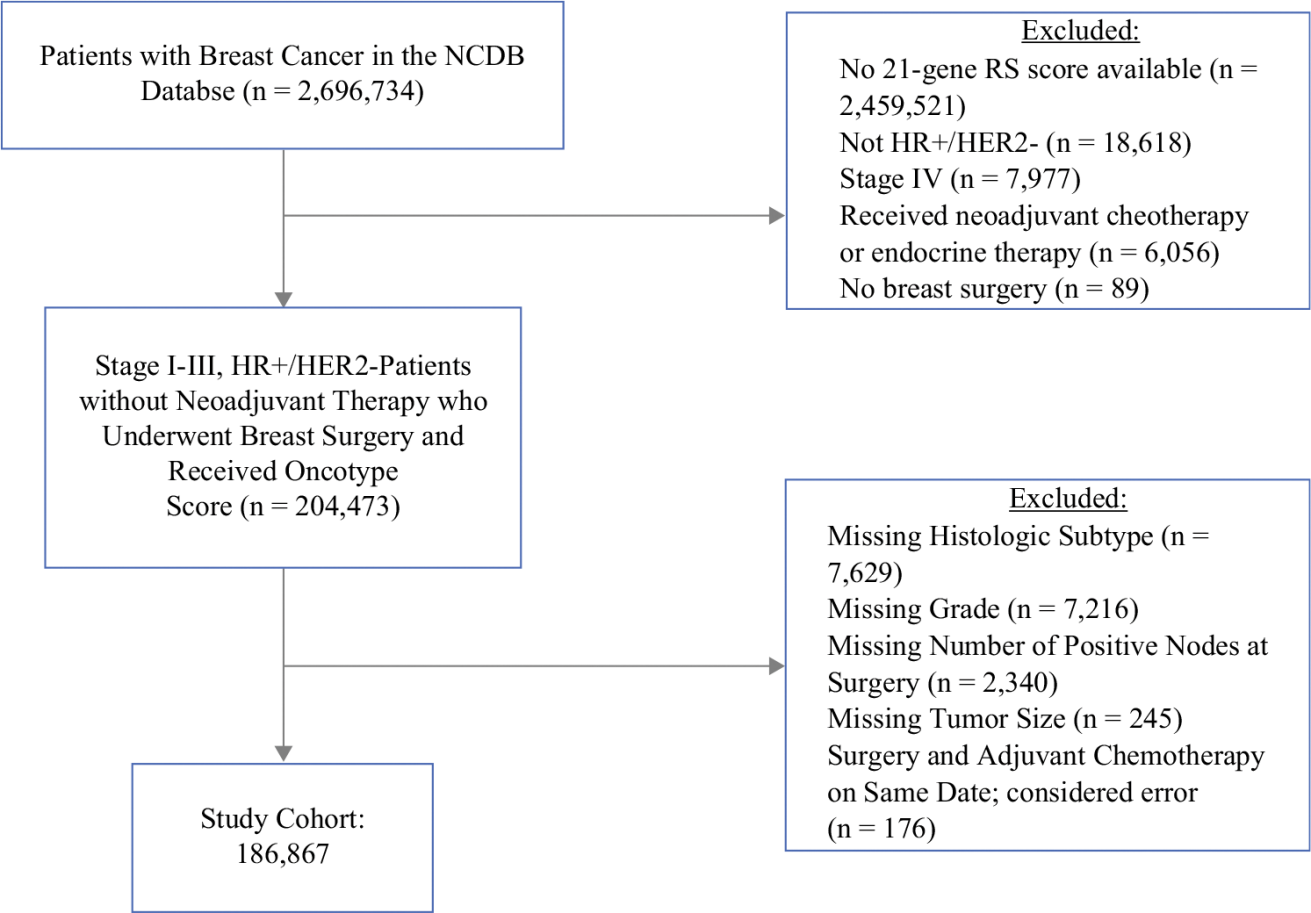
CONSORT diagram for study population. *CONSORT* Consolidated Standards of Reporting Trials, *NCDB* National Cancer Database, *RS* recurrence score, *HR*+ hormone receptor positive, *HER2*− human epidermal growth factor receptor 2-negative

A total of 2,696,734 patients with breast cancer were included in the original NCDB database. After excluding patients based on the criteria previously described, there were 186,867 patients with stage I–III, HR-positive, HER2-negative disease without neoadjuvant therapy who underwent breast surgery and received 21-gene RS testing who were included in our analysis (Fig. 1). Of these, 149,182 (79.8%) patients had IDC and 37,685 (20.2%) patients had ILC. Median follow-up time for the cohort was 36 months (IQR 23.1–59.0). The majority of patients had ER- and PR-positive cancers (*n* = 169,337, 90.6%), with 17,320 (9.27%) patients having ER-positive and PR-negative cancers.

### Invasive Lobular Carcinoma Versus Invasive Ductal Carcinoma Cohorts

Patients in the ILC cohort were slightly older than those in the IDC cohort (mean age 60.5 years vs. 58.9; *p* < 0.001). Additionally, they were more likely to present with higher stage disease and underwent mastectomy at higher rates (Table 1). The ILC tumors were significantly less likely to be grade 3, and overall chemotherapy was used less often in the ILC cohort compared with those with IDC. There was also a statistically significant difference in Charlson–Deyo score between the ILC and IDC cohorts, with ILC patients more likely to have a Charlson–Deyo score of 0 compared with those with IDC (85.0% vs.84.5%, *p* = 0.015).

### Clinical and Genomic Risk Discordance

We found a significantly higher rate of clinical high/genomic low risk status in patients with ILC compared with IDC. Patients with ILC were more likely to have high clinical risk by modified Adjuvant! Online than those with IDC (43% vs. 35.6%; *p* < 0.001) [Table 1]. Consistent with this finding, those with ILC were less likely to be node-negative than those with IDC (80.7% vs. 82.8%; *p* < 0.001). While clinical risk was higher in ILC patients, genomic risk by RS was much lower, with an incidence of RS >25 in ILC patients of 8.4% compared with 16.2% in IDC patients (*p* < 0.001). Together, this resulted in significantly higher rates of this discordant risk category, with 37.8% of the ILC group being clinical high/genomic low compared with 24.9% of the IDC group (*p* < 0.001) [Table 2].

Within the group of patients with 1–3 positive nodes, patients with ILC were significantly more likely to have an RS ≤25 compared with those with IDC (92.5% vs. 85.8%, respectively; *p* < 0.001) [Table 3]. This difference was more pronounced in those with >3 positive nodes, with the vast majority of ILC patients in this group (90.1%) having an RS ≤25 compared with 78.3% in the IDC group (*p* < 0.001) [Table 3]. Interestingly, among patients under the age of 50 years, ILC patients remained significantly more likely to have RS ≤25 than those with IDC across all nodal involvement categories. In patients under age 50 years with 1–3 positive nodes, low RS occurred in 94.8% of the ILC group versus 85.1% of the IDC group; among the small number of patients with >3 positive nodes, low RS occurred in 94.7% of the ILC group versus 70.6% of the IDC group (both *p* < 0.001 [Table 3].

### Chemotherapy Use

**FIG. 2:**
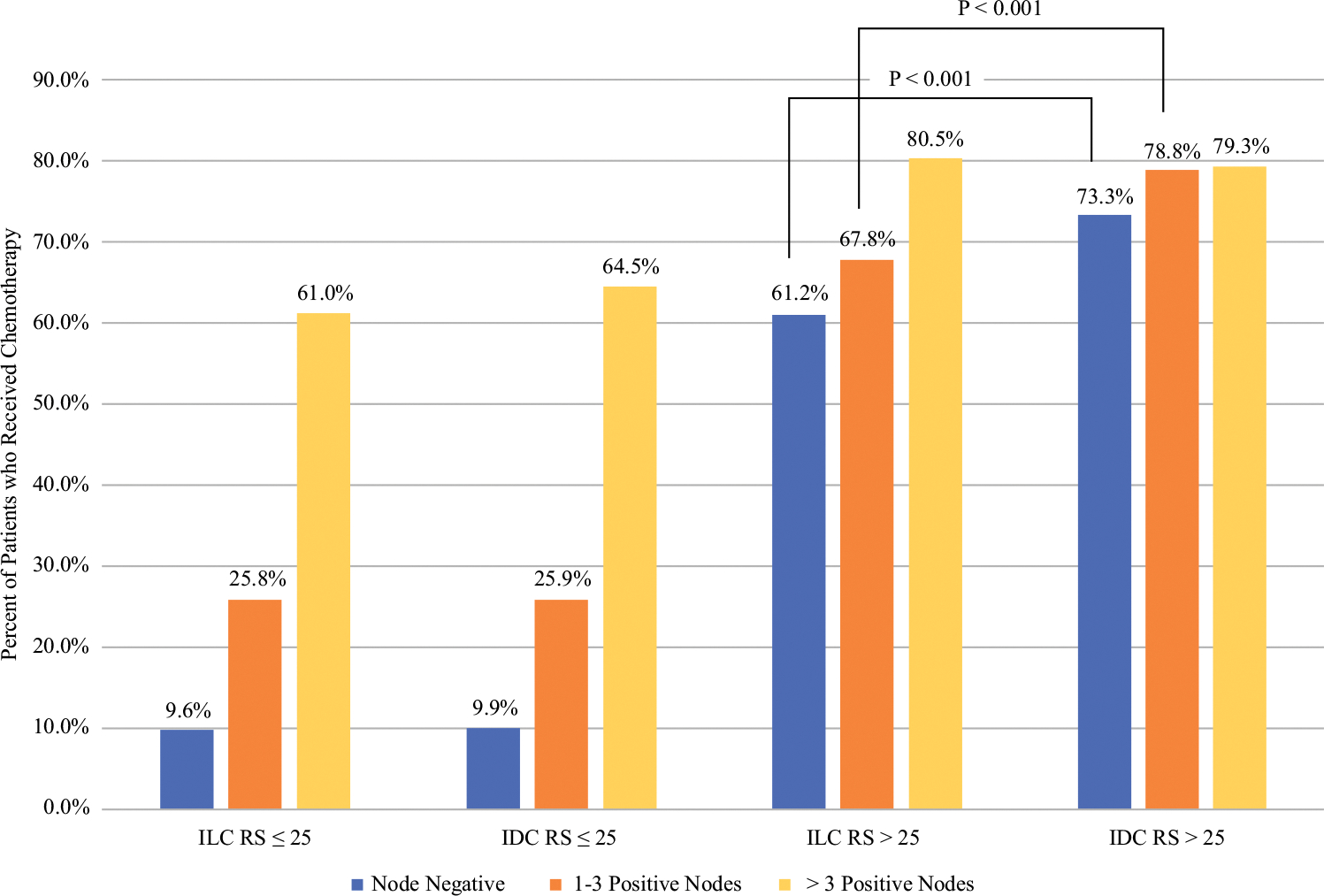
Proportion of patients who received chemotherapy, by histology, nodal status, and recurrence score. Chi-square test comparing patients with ILC/RS >25/node-negative vs. IDC/RS >25/node-negative and ILC/RS >25/1–3 positive nodes versus IDC/RS >25/1–3 positive nodes both yielded *p* < 0.001. All other comparisons were not statistically significant. *ILC* invasive lobular carcinoma, *IDC* invasive ductal carcinoma, *RS* recurrence score

We then evaluated the receipt of chemotherapy by histology, nodal status, RS category, and age. Among patients with RS ≤25, there was no difference in receipt of chemotherapy by histology across the node-negative, 1–3 positive nodes, and >3 positive nodal groups (Fig. 2). However, among patients with RS >25, those with ILC were significantly less likely to receive chemotherapy than those with IDC among patients with negative nodes or 1–3 positive nodes (both *p* < 0.001). In those with high clinical risk and RS >25, chemotherapy was used significantly more often in patients under 50 years of age compared with those aged 50 years or older, in both the ILC and IDC groups. However, among clinically high-risk patients with RS >25 and aged over 50 years, those with ILC were still less likely to receive chemotherapy than those with IDC (63.3% vs. 74.8%, *p* < 0.001). In ILC patients with high clinical risk and RS >25, 88.7% of patients were aged 50 years or older and 63.3% received chemotherapy compared with 80.5% of those under age 50 years (*p* < 0.001). Among IDC patients with high clinical risk and RS >25, 78.2% of patients were aged 50 years or older and 74.8% received chemotherapy compared with 88.2% of those under age 50 years (*p* < 0.001).

### Overall Survival Outcomes

**FIG. 3:**
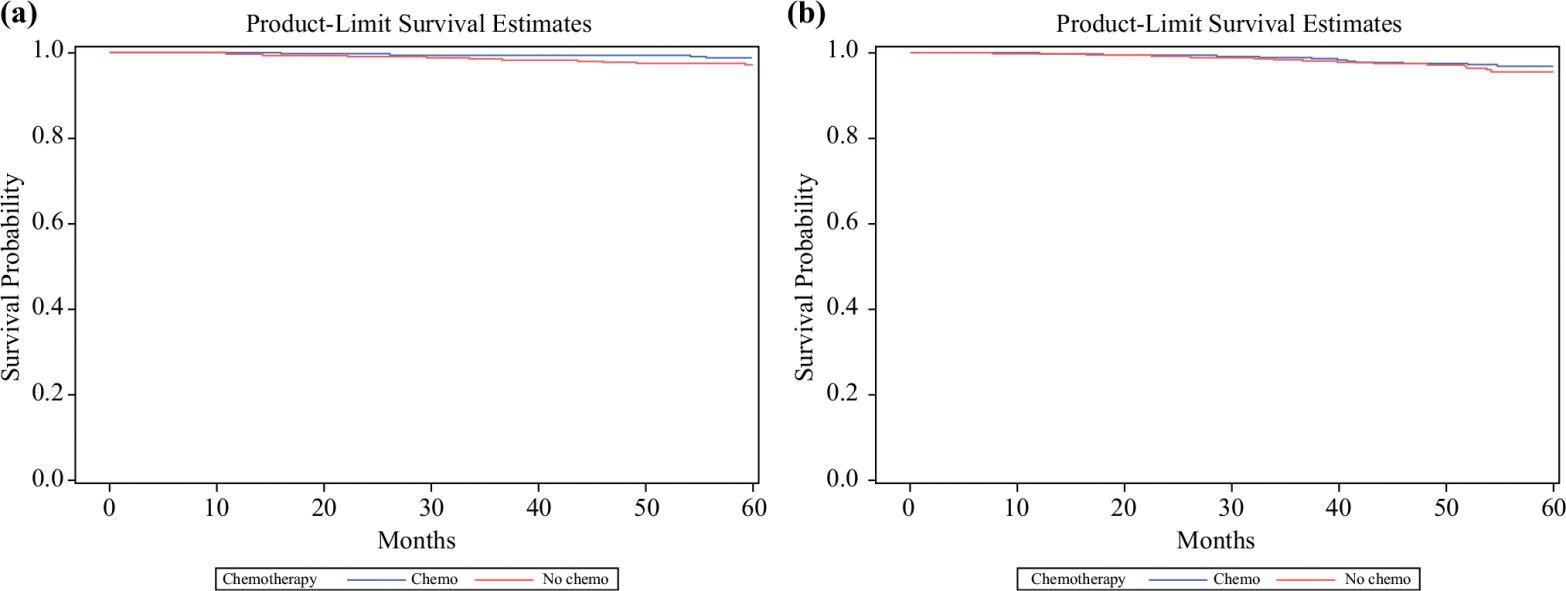
Propensity score-matched analysis using the greedy fixed matching method comparing overall survival in individuals who received chemotherapy compared with those who did not among patients with 1–3 positive nodes and RS ≤25, by histology. **a** For patients with IDC, survival between those who received chemotherapy and those who did not was not significantly different from one another (stratified log-rank test *p* = 0.278). **b** Similarly, for patients with ILC, survival between those who received chemotherapy and those who did not was not significantly different from one another (stratified log-rank test *p* = 0.532). *ILC* invasive lobular carcinoma, *IDC* invasive ductal carcinoma, *RS* recurrence score

Finally, we performed a propensity score-matched analysis to compare overall survival in individuals who received chemotherapy compared with those who did not, by histology among those with 1–3 positive nodes and RS ≤25, including patients of all ages. All covariates used in matching met the sample balance criteria, and baseline clinical and pathologic characteristics of the matched cohorts are provided in electronic supplementary Table 1. Among patients with IDC, there was no statistically significant difference in survival between those who did and did not receive chemotherapy (stratified log-rank test *p* = 0.278) [Fig. 3a]. Similarly, for patients with ILC, survival between those who received chemotherapy and those who did not was not significantly different from one another (stratified log-rank test *p* = 0.532) [Fig. 3b]. There were too few patients under the age of 50 years to perform a propensity score-matched analysis, therefore exploratory results from unmatched survival analyses are reported. For women under age 50 years, with 1–3 positive nodes and RS ≤25, unadjusted analysis showed that chemotherapy was associated with a significant improvement in overall survival for those with IDC (hazard ratio [HR] 0.44, 95% confidence interval [CI] 0.22–0.86; *p* = 0.016) but not for those with ILC (HR 0.54, 95% CI 0.14–2.18; *p* = 0.39). The association between chemotherapy and improved overall survival in those with IDC persisted when adjusted for Charlson–Deyo Index (HR 0.44, 95% CI 0.22–0.85; *p* = 0.016). There was no statistical interaction between chemotherapy and histology on overall survival.

## DISCUSSION

In this study of 186,867 patients with HR-positive, HER2-negative invasive breast cancer who underwent 21-gene RS testing in the NCDB, we found that patients with ILC have higher rates of discordant clinical and genomic risk than those with IDC. This finding is consistent with our prior work, which showed higher rates of discordant clinical and genomic risk in ILC patients using the 70-gene signature.^5^ Additionally, we found that ILC patients with 1–3 positive nodes were significantly more likely to have RS ≤25 compared with those with IDC, in both those under age 50 years and those aged 50 years or older.

Among those with RS ≤25, there was no difference in receipt of chemotherapy by histology regardless of nodal involvement. However, among those with RS >25, those with ILC were significantly less likely to receive chemotherapy than those with IDC among patients with negative nodes or 1–3 positive nodes. While these findings are consistent with our prior study, which showed lower rates of chemotherapy use in patients with high clinical risk ILC and high genomic risk as defined by 70-gene signature testing, they are nonetheless surprising given the general acceptance of chemotherapy in the setting of RS >25 and did not appear to be driven by older age in the ILC group.^5,14^ These findings may reflect hesitation on the part of clinicians and patients to utilize chemotherapy in ILC, where multiple studies have shown less benefit in the neoadjuvant and adjuvant settings.^22,23^ Together, this illustrates the treatment dilemma that clinicians and patients face. While patients with ILC are more likely to have high clinical risk, which portends increased risk of recurrence without chemotherapy, genomic assays and reported series suggest decreased chemotherapy benefit.^24^

We did not demonstrate any effect of chemotherapy on overall survival among patients with 1–3 positive nodes and RS ≤25 in our propensity score-matched analyses, regardless of histologic subtype. Interestingly, we did find an association between chemotherapy and overall survival in unmatched analysis of IDC patients under age 50 years with 1–3 positive nodes and RS ≤25, but not among those under age 50 years with ILC. Given the retrospective nature of this analysis, differences in patient selection for receiving chemotherapy likely contribute to chemotherapy-related outcomes. While we attempted to account for potential confounders in treatment selection by using propensity score matching by age at diagnosis, pathologic stage, and facility type, it is unlikely that we were able to fully adjust for potential bias between the chemotherapy and non-chemotherapy groups. Additionally, there may be a subgroup of patients with ILC within this cohort who may indeed benefit from chemotherapy. As such, this question should remain open and more research is needed.

This bias is greater in our exploratory unmatched analysis in patients under age 50 years, which showed a significant improvement in overall survival among IDC patients who received chemotherapy compared with those who did not, but no difference in those with ILC. There are several potential explanations for the findings of our exploratory analyses. One possibility is that among those under age 50 years with 1–3 positive nodes and RS ≤25, the overall survival difference observed in the IDC group resulted from patient selection bias and not chemotherapy effect. Another explanation is that we were unable to detect an overall survival benefit in ILC patients under age 50 years, with 1–3 positive nodes and RS ≤25 because of small numbers in this subgroup. Lastly, it is possible that chemotherapy improves overall survival in IDC patients but not ILC patients under the age of 50 years with 1–3 positive nodes and RS ≤25; for this reason, reporting of long-term and overall survival outcomes by histologic subtype from trials such as RxPONDER is needed.

While the RxPONDER trial showed a significant improvement in invasive disease-free survival in patients under age 50 years who had 1–3 positive nodes and RS ≤25 who received chemotherapy, we cannot directly compare our results since the NCDB lacks recurrence endpoints. It is important to note that for patients with HR-positive/HER2-negative tumors, and ILC especially, recurrence events and consequently impact on overall survival can happen at later timepoints, highlighting the need for longer term follow-up.^25,26^ Additionally, we lacked data on type of endocrine therapy, which likely impacts outcomes. Finally, other limitations of our study include lack of information regarding type of chemotherapy regimen, adherence to endocrine therapy, and short follow-up time for patients included in our study.

This study has many strengths, including the use of a relatively large number of ILC patients, and is now the second study to demonstrate that patients with ILC have high rates of discordance between clinical and genomic risk based on widely used molecular assays. While many studies show that ILC tumors have lower response rates to chemotherapy in the neoadjuvant setting, and less benefit from chemotherapy in the adjuvant setting, there may still be a subset of chemotherapy-sensitive ILC cases. Recent work has identified genomic signatures that identify subtypes within ILC, suggesting heterogeneity within this tumor type.^27,28^ The high incidence of high clinical risk among patients with ILC highlights the need for both more effective therapies, and potentially ILC-specific prediction tools. More broadly, improving outcomes for these patients with ILC will require not only equitable enrollment of ILC patients into breast cancer clinical trials but also histologic subtype-specific reporting of trial results.

## Supplementary Material

1838712_Sup_Material

## Figures and Tables

**TABLE 1:** Clinicopathologic characteristics of the study cohort

	ILC [*n* = 37,685]	IDC [*n* = 149,182]	*p*-Value

Age at diagnosis, years [mean (SD)]	60.5 (10.0)	58.9 (10.6)	<0.001
Age <50 years	31,597 (83.9)	117,985 (79.1)	
*Pathologic stage*			<0.001
I	21,384 (56.7)	104,748 (70.2)	
II	15,302 (40.6)	43,194 (29.0)	
III	999 (2.7)	1240 (0.8)	
*Nodal involvement*			<0.001
Node-negative	30,401 (80.7)	123,470 (82.8)	
1–3 positive	6869 (18.2)	24,919 (16.7)	
>3 positive	415 (1.1)	793 (0.5)	
*Tumor grade*			<0.001
1	9451 (25.1)	43,276 (29.0)	
2	25,403 (67.4)	78,871 (52.9)	
3	2831 (7.5)	27,035 (18.1)	
*Clinical risk*			<0.001
Low	21,464 (57.0)	96,060 (64.4)	
High	16,221 (43.0)	53,122 (35.6)	
Genomic risk	34,534 (91.6)	125,030 (83.8)	<0.001
21-gene RS ≤25	3151 (8.4)	24,152 (16.2)	
21-gene RS >25			
*Surgical therapy*			<0.001
Lumpectomy	21,373 (56.7)	102,467 (68.7)	
Mastectomy	16,312 (43.3)	46,715 (31.3)	
*Adjuvant therapy*			
Chemotherapy	6383 (17.3)	33,534 (22.9)	<0.001
Endocrine therapy	35,161 (94.6)	137,003 (93.4)	<0.001
*Charlson–Deyo score*			0.013
0	32,039 (85.0)	126,007 (84.5)	
1	4629 (12.3)	18,834 (12.6)	
2	799 (2.1)	3312 (2.2)	
≥3	218 (0.6)	1029 (0.7)	

Data are expressed as *n* (%) unless otherwise specified

*ILC* invasive lobular carcinoma, *IDC* invasive ductal carcinoma, *RS* recurrence score, *SD* standard deviation

**TABLE 2:** Distribution of clinical and genomic risk categories by histology. Genomic risk was categorized as either 21-gene RS ≤25 or >25

	ILC [*n* = 37,685]	IDC [*n* = 149,182]	*p*-Value^a^	*p*-Value^b^

Clinical high/21-gene RS ≤25	14,253 (37.8)	37,126 (24.9)	<0.001	<0.001
Other	23,432 (62.1)	112,056 (75.1)		
Clinical low/21-gene RS ≤25	19,601 (52.0)	85,078 (57.0)		
Clinical high/21-gene RS >25	1968 (5.2)	15,996 (10.7)		
Clinical low/21-gene RS >25	1863 (4.9)	10,982 (7.4)		

*ILC* invasive lobular carcinoma, *IDC* invasive ductal carcinoma

a *p*-value from Chi-square comparing clinical high/genomic low compared with other risk categories combined

b *p*-value from Chi-square comparing across all four risk categories

**TABLE 3:** Breakdown of nodal status by histology and 21-gene recurrence score in all patients (top) and for those with age < 50 years (bottom)

	ILC 21-Gene RS ≤25 [*n* (%)]	IDC 21-Gene RS ≤25 [*n* (%)]	*p*-Value^a^

*All patients*			
Node-negative	27,804 (91.5)	103,039 (83.5)	<0.001
1–3 positive nodes	6356 (92.5)	21,370 (85.8)	<0.001
>3 positive nodes	374 (90.1)	621 (78.3)	<0.001
*Patients aged <50 years*			
Node-negative	4644 (94.6)	22,016 (84.6)	<0.001
1–3 positive nodes	1062 (94.8)	4,251 (85.1)	<0.001
>3 positive nodes	54 (94.7)	115 (70.6)	<0.001

*ILC* invasive lobular carcinoma, *IDC* invasive ductal carcinoma, RS recurrence score

a *p*-value from Chi-square comparing ILC 21-gene RS ≤25 and 21-gene IDC RS ≤25 across nodal categories

## Data Availability

The data that support the findings of this study are available from the NCDB, but restrictions apply to the availability of these data, which were used under license for the current study and therefore are not publicly available. Data are however available from the authors upon reasonable request and with permission of the NCDB.
