# Supplementary material for: The 21-Gene Recurrence Score in Clinically High-Risk Lobular and Ductal Breast Cancer: A National Cancer Database Study
Source: Ann Surg Oncol. Author manuscript; Available in PMC 2022 Nov 1. (PMC9550696; doi:10.1245/s10434-022-12065-3)
Supplement: 1838712_Sup_Material [file NIHMS1838712-supplement-1838712_Sup_Material.docx]

**Supplementary Table 1:** Clinicopathologic characteristics of matched cohort used in the propensity score matching analysis (all with 21-gene Recurrence Score $\boldsymbol{\leq}$ 25 and 1-3 positive nodes)

|  | ILC  (N = 2,628) | IDC  (N =8,726 ) | P-Value |
| --- | --- | --- | --- |
| Age at diagnosis (years), mean (SD)  Age < 50 years | 56.5 (9.0)  686 (26.1) | 55.9 (9.1)  2,502 (28.7) | 0.003  0.010 |
| Pathologic stage, n (%)  I  II  III | 305 (11.6)  2,080 (79.1)  243 (9.2) | 1,473 (16.9)  7,109 (81.5)  144 (1.7) | <0.001 |
| Tumor grade, n (%)  1  2  3 | 625 (23.8)  1,826 (69.5)  177 (6.7) | 2,506 (28.7)  5,058 (58.0)  1,162 (13.3) | <0.001 |
| Clinical risk, n (%)  Low  High | 322 (12.3)  2,306 (87.7) | 1,871 (21.4)  6,855 (78.6) | <0.001 |
| Surgical therapy, n (%)  Lumpectomy  Mastectomy | 1,127 (42.9)  1,501 (57.1) | 5,055 (57.9)  3,671 (42.1) | <0.001 |
| Adjuvant therapy, n (%)  Chemotherapy  Endocrine Therapy | 1314 (50.0)  2,481 (95.6) | 4,363 (50.0)  8,232 (95.3) | >0.99  0.56 |
| Charlson-Deyo Score, n (%)  0  1  2  ≥3 | 2,261 (86.0)  306 (11.6)  47 (1.8)  14 (0.5) | 7,440 (85.3)  1,062 (12.2)  173 (2.0)  51 (0.6) | 0.78 |

**Legend**: ILC = invasive lobular carcinoma; IDC = invasive ductal carcinoma; RS = recurrence score
